# Taming the beast: are multidisciplinary Endocarditis Teams enough?

**DOI:** 10.1007/s12471-022-01751-2

**Published:** 2022-12-12

**Authors:** R. K. Riezebos, R. Cocchieri

**Affiliations:** 1 Department of Cardiology, Heart Centre, OLVG Hospital, Amsterdam, The Netherlands; 2 Department of Cardiothoracic Surgery, Heart Centre, OLVG Hospital, Amsterdam, The Netherlands

Endocarditis is a serious and often fatal disease in which the diagnosis is often difficult to make. The first descriptions of endocarditis date from the 17th century. The clinical picture was first described by the Frenchman Lazare Rivière who was the personal physician of King Louis the 13th. At that time, a diagnosis of endocarditis was considered a death sentence. Today, we know that early recognition and treatment is crucial for the prevention of complications such as heart failure, conduction disorders, and embolisation.

**Fig. 1:**
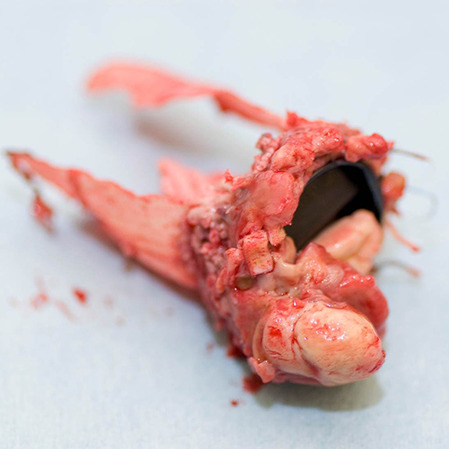
Explanted Bentall prosthesis with mechanical bi-leaflet aortic valve that shows a large vegetation on the ventricular side

In the last 50 years, several changes have transformed the epidemiological patterns of endocarditis: the average age of patients has increased, it is now more common in men, and there is a higher proportion of prosthesis-related endocarditis due to the increasing use of implants. In recent decades, the microbiological pattern of endocarditis has also changed. Apparently, the *Staphylococcus aureus* is becoming increasingly more common. This bacterium often presents with an acute and severe illness that leads to early destruction of valve tissue and carries a higher risk for embolisation (see Fig. 1; [1, 2]).

Treatment of endocarditis is challenging. Despite guidelines that include diagnostic flow charts and antibiotic therapy advice, there appears to be quite some practice variation. This also applies to surgical treatments where a significant variation is observed in indication and timing of surgery [3]. As a result, the European Society of Cardiology (ESC) advises to formally install dedicated Endocarditis Teams in specialised hospitals. Patients with uncomplicated endocarditis can be treated in a non-referral centre, from where early and frequent communication with the Endocarditis Team of the reference centre is mandatory. Patients with complicated endocarditis should be evaluated and treated at a tertiary centre, which has the availability of immediate heart surgery and where an Endocarditis Team consisting of an infectiologist, microbiologist, cardiologist, imaging specialist and a cardiac surgeon is on site [4].

In a prospective evaluation of the Endocarditis Team by Wahadat et al. in this edition of the *Netherlands Heart Journal*, the added value of the Endocarditis Team at a tertiary referral centre is demonstrated [5]. This E‑team meets with a high regularity and consists of an extensive multidisciplinary group of professionals. The diagnosis using the modified Duke criteria was improved by means of advanced diagnostic advice in a significant number of patients. The guidance of the Endocarditis Team frequently resulted in adjustment of the antibiotic therapy. However, in only 5% of cases it was advised to perform surgery in patients for whom an earlier conservative strategy had been decided. This and other studies cautiously conclude that mortality could be reduced by the proper implementation of Endocarditis Teams [6–8].

However, it remains disappointing that the very high mortality rate of endocarditis prevails despite multidisciplinary treatment teams, modern drug therapy and surgical possibilities. Given the outcomes of the disease, one may wonder whether there is such a thing as an uncomplicated endocarditis. In recent years, a collaboration guided by the Netherlands Heart Institute (NHI) has started an initiative to promote multidisciplinary collaboration, research, and education. To gain more insight into the screening, diagnostics, treatment and outcomes of endocarditis in the Netherlands, a prospective registry will be initiated. This *Endocor* registry will start in several referral centres in early 2023 and will be rolled out over all Dutch Heart Centres in the next few years.

Important questions on the subject include the duration and method of administration of antibiotics. The Danish POET trial has shown that a step-down therapy with oral antibiotics after clinical stabilisation of patients with endocarditis was at least noninferior to continued intravenous antibiotic therapy [9]. Yet, this promising strategy is hardly applied in clinical practices. There has also been much debate about patient selection for and timing of surgery. This is of particular importance, because it seems that elderly—and especially elderly women—are operated on less often and that this is accompanied by a worse prognosis [10]. These results state the importance of razor-sharp recognition of a surgical indication and of an improved surgery performance in elderly patients. A wider adoption of a minimally invasive approach, even in urgent cases, could benefit this specific frail group and should be advocated.

Due to the increase in aging population and the further development of invasive treatments such as cardiovascular implants, the epidemiology of endocarditis will further change in the upcoming decades. Our patients will be even more elderly and more fragile, and the percentage of prosthesis and device endocarditis will continue to increase. Therefore, in the short-term and medium-term the prognosis of the disease is not expected to improve. However, rapid—and adequate—diagnostics, state-of-the-art surgical treatment including minimally invasive access and individualised antibiotic therapy provide the best chances to combat endocarditis. To achieve the optimal workflows, treating endocarditis patients in specialised high-volume centres should be considered. In addition, the multidisciplinary Endocarditis Teams could include geriatricians to best serve the increasing number of elderly patients with the disease. And finally, good, and complete registration will aid to the improvement of the multilevel therapy that the disease requires. Step by step, we will have greater insight into how to tame this multi-faceted monster of a disease.
